# Surgical Treatment for Bacteremia due to Translocation of *Enterococcus gallinarum*: A Case Report

**DOI:** 10.70352/scrj.cr.25-0686

**Published:** 2026-03-18

**Authors:** Yoshitaka Imoto, Masato Yamadera, Tomohiro Tsuru, Koichi Okamoto, Yoshiki Kajiwara, Yoji Kishi, Hideki Ueno

**Affiliations:** Department of Surgery, National Defense Medical College, Tokorozawa, Saitama, Japan

**Keywords:** *Enterococcus gallinarum*, bacterial translocation, colectomy

## Abstract

**INTRODUCTION:**

Bacterial translocation (BT) followed by bacteremia is usually managed with antibiotics, but some cases remain refractory. Herein, we report an immunocompromised patient with bacteremia due to the translocation of *Enterococcus gallinarum*, a rare pathogen among enterococcal infections, who was successfully treated with surgical intervention.

**CASE PRESENTATION:**

A 69-year-old woman with prior low anterior resection for rectal cancer developed adhesive intestinal obstruction during steroid treatment for suspected pyoderma gangrenosum. She progressed to septic shock despite antibiotics. CT revealed an edematous, thickened colon, suggesting ischemia or necrotizing colitis. An urgent laparotomy revealed no necrosis. The markedly edematous right colon was resected, with the creation of an ileostomy and a mucous fistula. Blood and colonic wall cultures both yielded *Enterococcus gallinarum*, confirming BT sepsis. Intravenous levofloxacin and selective digestive decontamination from the mucous fistula achieved recovery. Stoma closure was performed 6 months later without recurrence of bacteremia.

**CONCLUSIONS:**

Intestinal resection serving as the portal of entry for BT should be considered in patients unresponsive to conservative management.

## Abbreviations

BT
: bacterial translocation
*E. gallinarum*
: *Enterococcus gallinarum*

## INTRODUCTION

Bacterial translocation (BT) is the passage of bacteria or endotoxins from the gut through the intestinal mucosa to other organs.^1)^ The intestinal mucosal barrier and gut microbiota are crucial in preventing pathogen invasion, but may be impaired in intestinal obstruction, malignant neoplasms, immunocompromised states, or after microbiota disruption by antibiotics.^2–5)^ Occult bacteremia may occur asymptomatically in healthy individuals as a result of BT. In immunocompromised patients, however, bacteremia can become clinically manifest, often posing therapeutic challenges and occasionally progressing to severe conditions.^6)^

Species causing BT include *Escherichia coli*, *Klebsiella pneumoniae*, *Klebsiella oxytoca*, *Enterobacter cloacae*, and *Enterococcus faecalis*.^7–9)^ Enterococci are Gram-positive, facultative anaerobic bacteria that constitute a part of the normal human gut flora. They may also be isolated from cultures of bile or intra-abdominal drains.^10,11)^ However, they can lead to infectious diseases such as urinary tract infections, endocarditis, meningitis, and catheter-related bloodstream infections. The common species of enterococci isolated from clinical specimens are *Enterococcus faecalis* and *Enterococcus faecium*. The incidence of *Enterococcus gallinarum* (*E. gallinarum*) is very low, at approximately 1%.^12–14)^ Herein, we report a case of bacteremia caused by translocation of *E. gallinarum* that needed emergent surgical intervention.

## CASE PRESENTATION

A 69-year-old woman was admitted for evaluation of a painful right leg ulcer persisting for approximately 1 year to the Department of Dermatology at our hospital. Her medical history included hypertension and intracerebral hemorrhage. She had undergone a low anterior resection for rectal cancer 7 years earlier. She was a regular smoker (20 pack-years) and social drinker. The hepatitis C virus antibody test was positive, and the ribonucleic acid test was negative. The patient was initially treated with prednisolone (30 mg/day) for suspected pyoderma gangrenosum. The skin ulcer was almost completely epithelialized, and the prednisolone dose was gradually tapered.

**Fig. 1:**
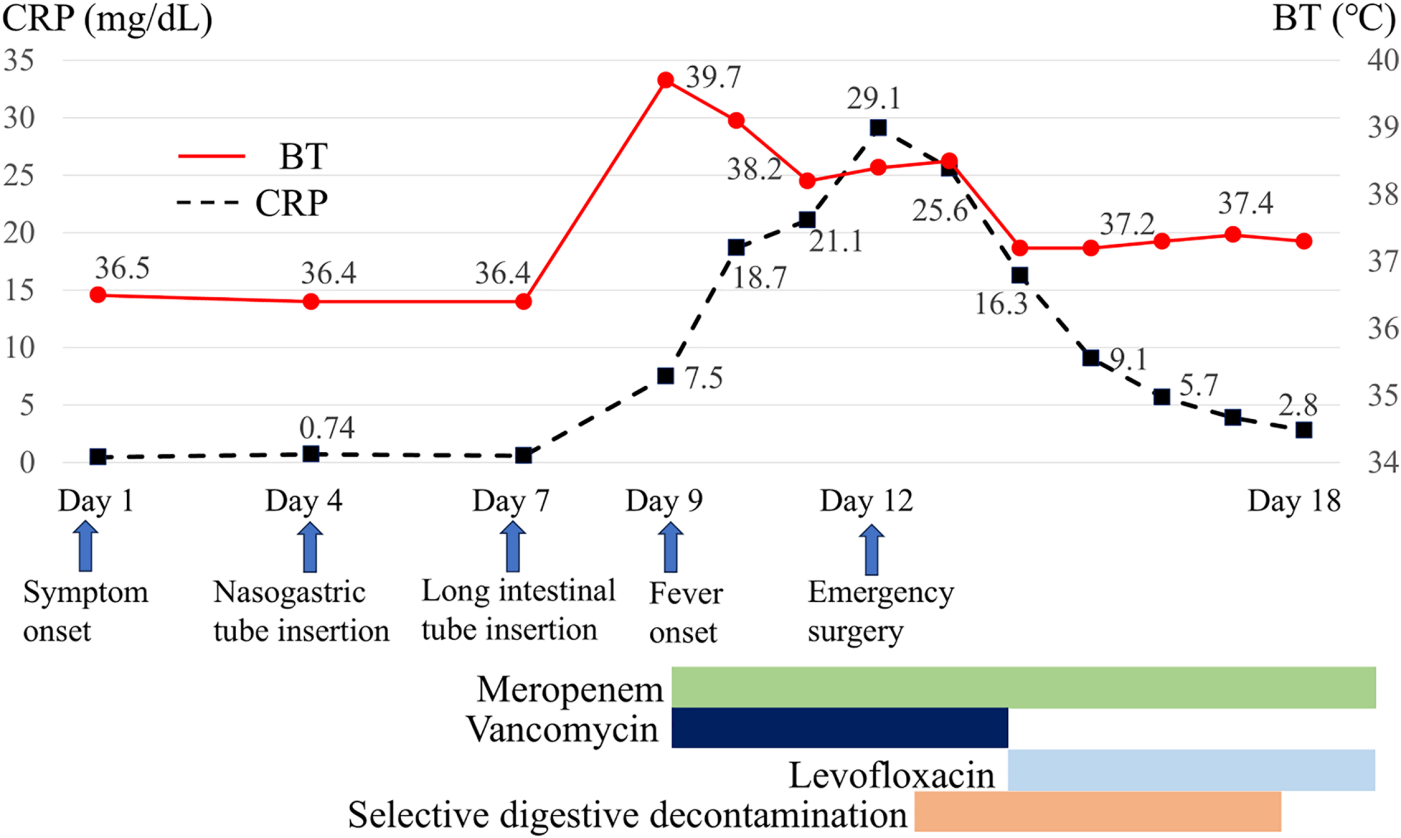
Clinical course of the patient. BT, body temperature; CRP, C-reactive protein

**Fig. 2:**
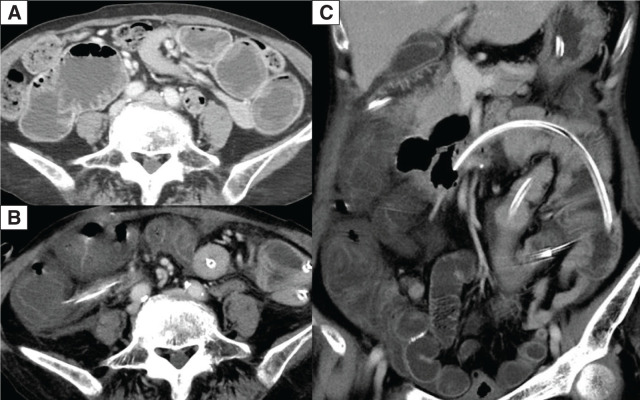
Contrast-enhanced CT images. (**A**) Axial image obtained at the disease onset, showing dilatation of the small intestine. (**B**, **C**) Preoperative images obtained during septic shock, showing that the colon is markedly edematous and slightly contrast-enhanced (**B**, axial view; **C**, coronal view).

After 1 month of prednisolone therapy, during tapering to 10 mg/day, she experienced multiple episodes of nausea and vomiting (**Fig. 1**). Physical examination showed abdominal distention. CT revealed a dilated and fluid-filled small intestine (**Fig. 2**). The patient was initially diagnosed with adhesive small bowel obstruction and managed conservatively with nasogastric decompression, but had persistent symptoms without improvement. A long intestinal tube was inserted for small intestine decompression 6 days after the onset. However, nausea and fatigue worsened, and a fever developed 2 days later, despite a decrease in the volume of long tube drainage and an improvement in abdominal distension. Physical examination revealed a body temperature of 38.8°C, blood pressure of 78/54 mmHg, and heart rate of 128 beats/min. Blood tests showed a white blood cell count of 6200/µL, C-reactive protein level of 7.47 mg/dL, and procalcitonin level of 22.67 ng/mL. She was treated with vancomycin (2g/day) and meropenem (3g/day) for suspected catheter-related bloodstream infection or cellulitis of the right lower leg as empiric therapy after bacterial examination. Five days after long tube insertion, her body temperature was 38.4°C, blood pressure was 99/67 mmHg, heart rate was 147 beats/min, and respiratory rate was 28 breaths/min. Findings on follow-up blood tests were: white blood cell count, 4100/µL; C-reactive protein, 29.1 mg/dL; procalcitonin, 44.0 ng/mL; albumin, 1.4 g/dL; creatinine, 1.1 mg/dL; prothrombin time, 65.2%; activated partial thromboplastin time, 40.5 s; fibrin/fibrinogen degradation product, 45 µg/mL; and antithrombin 52%. Follow-up CT revealed a markedly edematous and thickened colon (**Fig. 2**). Although CT did not demonstrate pneumatosis intestinalis or portal venous gas, the patient was in septic shock requiring noradrenaline; therefore, non-occlusive mesenteric ischemia or gangrenous ischemic colitis was clinically suspected. Given the suspicion of impaired colonic perfusion, bowel resection had been planned preoperatively, and an emergency laparotomy was performed.

**Fig. 3:**
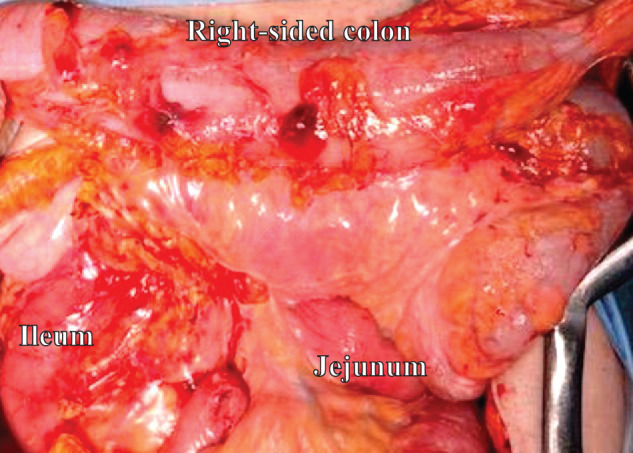
Intraoperative findings. The intestine, especially the right-sided colon, was edematous.

During laparotomy, the right colon was markedly edematous without overt necrosis or perforation (**Fig. 3**). As originally planned preoperatively, the right-sided colon, which exhibited the most severe edema, was resected. A single-hole ileostomy was created, and the distal side of the transverse colon was constructed as a mucous fistula.

**Fig. 4:**
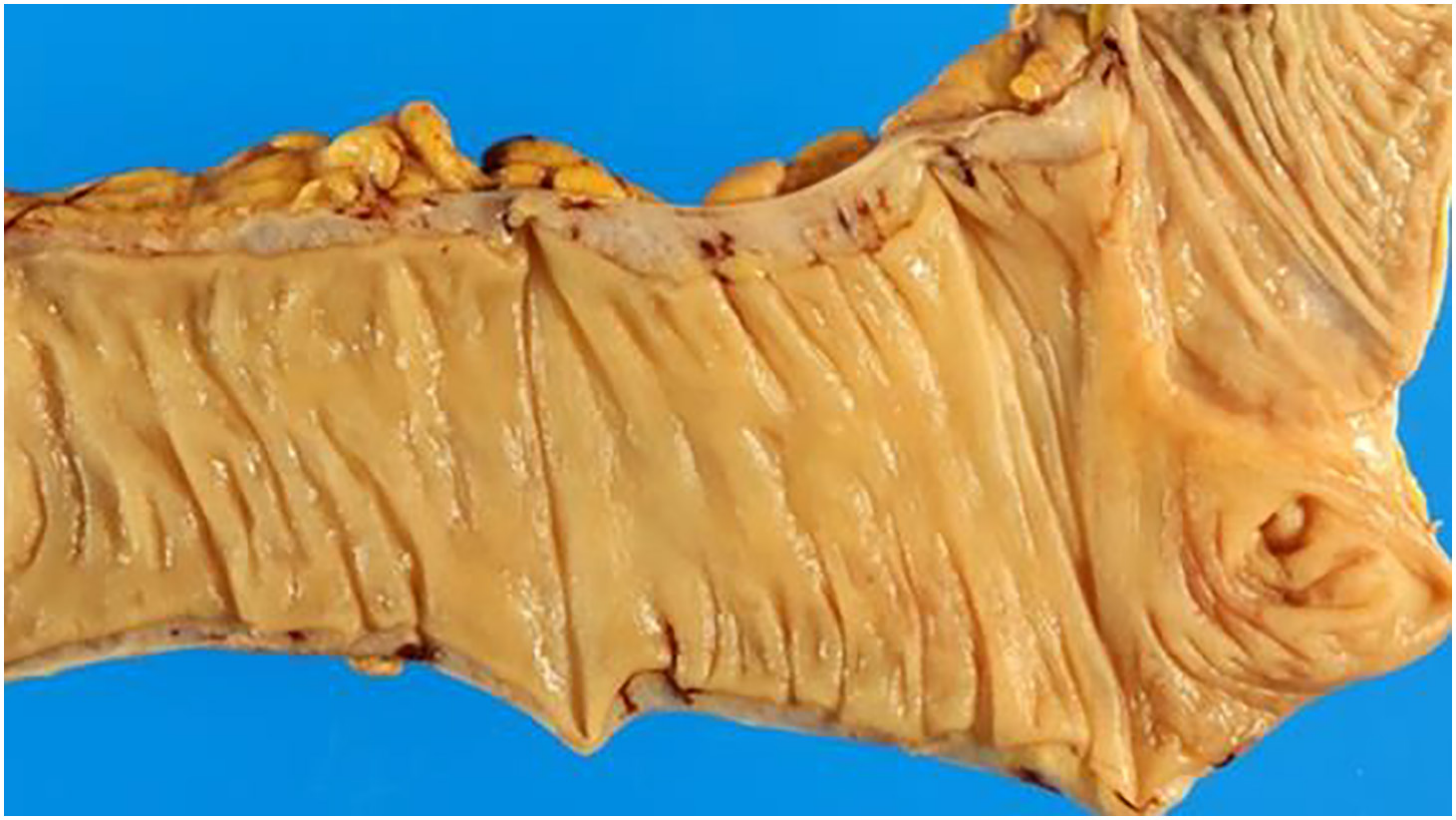
The resected specimen shows that the colon is edematous but not necrotic.

**Fig. 5:**
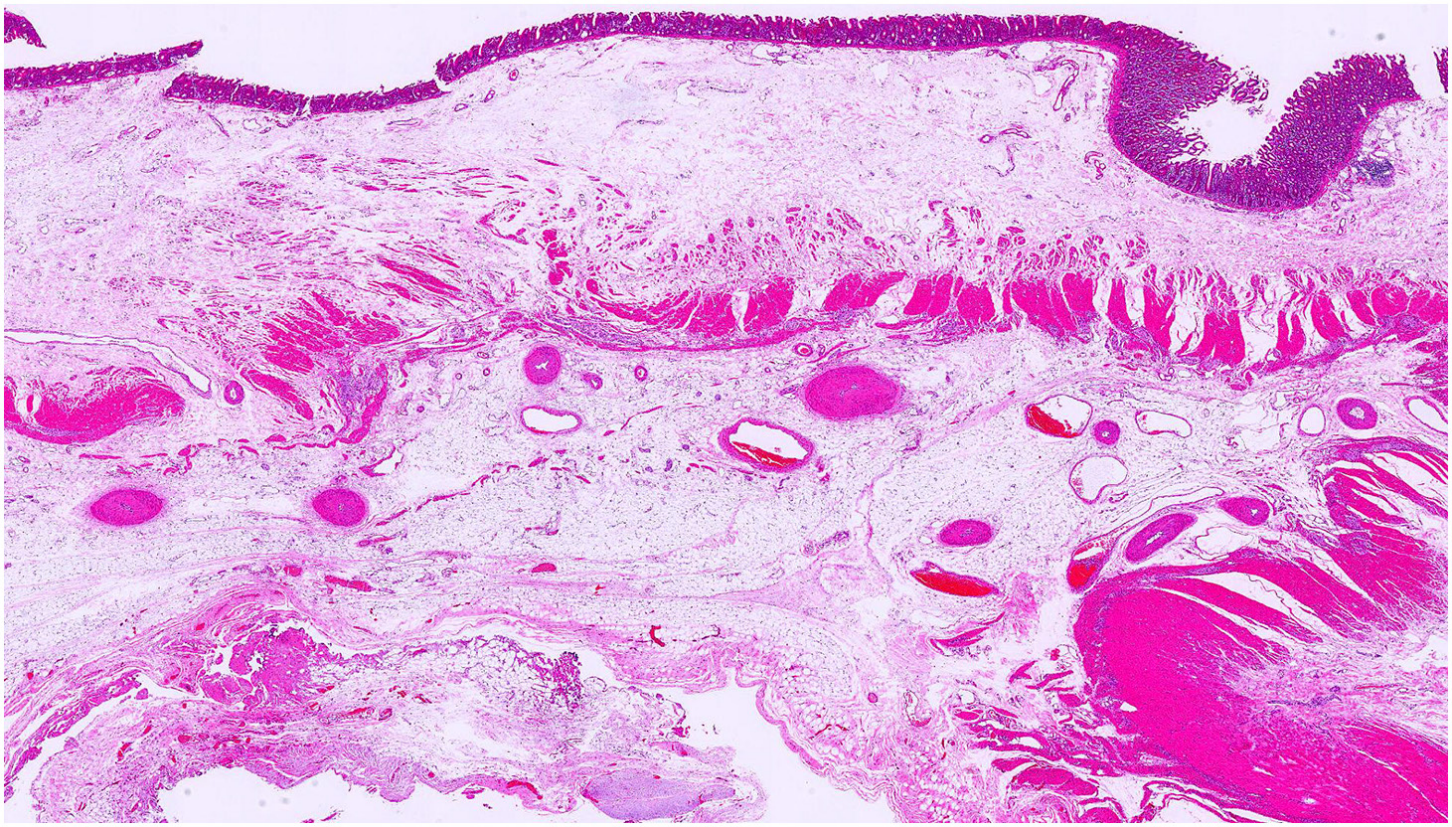
Histopathological examination shows edematous changes in the intestine (Hematoxylin-eosin staining).

The resected specimen was macroscopically markedly edematous (**Fig. 4**). No mucosal abnormalities, such as ulcers or malignant tumors, were observed from the pathological examination (**Fig. 5**).

Culture from the leg ulcer yielded methicillin-resistant *Staphylococcus aureus*. The results of the blood cultures obtained preoperatively became available postoperatively, revealing the presence of *K. oxytoca*, *Citrobacter freundii*, and *E. gallinarum*. The culture of the resected intestinal mucosa specimen yielded *E. gallinarum*, which was consistent with the blood culture results. Thus, we diagnosed bacteremia due to the translocation of *E. gallinarum*. Based on antimicrobial susceptibility testing, *K. oxytoca* and *C. freundii* were resistant to ampicillin and susceptible to meropenem, whereas *E. gallinarum* was resistant to vancomycin and susceptible to ampicillin and levofloxacin. The susceptibility category was not provided because interpretive breakpoints for meropenem against *E. gallinarum* have not been established.

On POD 2, the culture results and antimicrobial susceptibility data became available, and the antimicrobial regimen was changed from meropenem and vancomycin to meropenem and levofloxacin. In addition, a selective digestive decontamination regimen, consisting of amphotericin B, polymyxin E, and vancomycin, was administered through the mucous fistula of the transverse colon into the remnant colorectum and was continued until POD 5. The patient did not develop any major postoperative complications. She was treated with prednisolone for pyoderma gangrenosum again and was transferred to a rehabilitation hospital 1 month after surgery. Six months after surgery, stoma closure was performed, and the postoperative course was uneventful without bacteremia recurrence.

## DISCUSSION

Generally, the standard treatment for bacteremia due to BT is antibiotics. To the best of our knowledge, this is the first reported case of surgical treatment for translocation of *E. gallinarum*. In the present case, surgery was beneficial not only for the treatment of *E. gallinarum-*associated BT sepsis but also for identification of the causative pathogen. Resection of the edematous colon was beneficial for infection control, as the resected specimen represented the portal of entry for BT. Furthermore, the isolation of the same organism, *E. gallinarum*, from both the resected colonic specimen and blood cultures confirmed the diagnosis of *E. gallinarum*-associated sepsis.

The initial nausea and vomiting were due to adhesive small bowel obstruction. However, BT was likely to have occurred in the right-sided colon rather than at the site of small bowel obstruction. This is supported by the fact that the volume of long tube drainage had been decreasing and abdominal distension was improving at the time of fever onset, whereas the right-sided colon exhibited the most severe edema. Although the pathological findings revealed no necrotic changes, marked colonic edema was evident. Severe colonic edema is capable of disrupting epithelial integrity and increasing permeability, thereby impairing the mucosal barrier and facilitating BT.^15–17)^

A report indicated that *E. gallinarum* infection is associated with polymicrobial bacteremia.^18)^ In the present case, polymicrobial bacteremia involving *K. oxytoca* and *C. freundii* was observed. However, based on the antimicrobial susceptibility results and the concordance between the intestinal wall and blood culture findings, *E. gallinarum* was the primary causative pathogen. These two organisms, similar to *E. gallinarum*, may enter the bloodstream via BT from the edematous colon. The two organisms were also potential contributors to BT-associated sepsis; however, susceptibility testing revealed that both organisms were susceptible to meropenem. By contrast, *Enterococcus* species generally exhibit low susceptibility to meropenem^19)^ and a susceptibility category was not available because interpretive criteria for meropenem against *E. gallinarum* have not been established. In addition, *E. gallinarum* is intrinsically resistant to vancomycin^20)^ and therefore was not covered by the combination of meropenem and vancomycin. This likely explains the deterioration of the patient’s preoperative vital signs and laboratory findings despite broad-spectrum antibiotic therapy. Notably, the identical organism with the same antimicrobial susceptibility profile was isolated from both blood cultures and intestinal mucosal cultures. Based on these findings, *E. gallinarum* was the primary causative pathogen of BT-associated sepsis, while the other organisms were regarded as secondary. At our institution, routine cultures of intestinal fluid or stool are performed only to confirm the absence of vancomycin-resistant enterococci and methicillin-resistant *S. aureus*. Because all three organisms were identified postoperatively, it could not be confirmed whether these organisms were present in the long tube drainage culture obtained preoperatively.

Surgical intervention is indicated in certain cases of severe colitis or intestinal ischemia, including *E. coli* O157:H7 infectious colitis,^21)^ fulminant amoebic colitis, nonocclusive mesenteric ischemia,^22)^ and gangrenous ischemic colitis.^23)^ Emergency surgery was performed due to antibiotic-refractory septic shock, with suspected nonocclusive mesenteric ischemia or gangrenous ischemic colitis based on laboratory and CT findings. The right-sided colon, including the ileocecal region, showed the most severe edema and was suspected of ischemia on CT; therefore, it was resected. Because the patient was immunocompromised due to steroid therapy and colonic edema alone can cause BT, colectomy was considered necessary for adequate infection control. Given that the surgery was performed on an emergency basis, indocyanine green and a fluorescence imaging system were not readily available at that time. In addition, the patient was in septic shock requiring noradrenaline, which might have resulted in weak or unreliable indocyanine green fluorescence.

There was a time lag of 2 days between surgery and the identification of levofloxacin as the appropriate antibiotic based on culture results. Levofloxacin was initiated on POD 2 and contributed to clinical improvement; however, inflammatory markers had already begun to improve before the initiation of levofloxacin. While bowel resection in the setting of septic shock remains controversial, adequate source control was essential in this case, and antibiotic therapy alone would likely have been insufficient. These findings indicate that right hemicolectomy contributed significantly to the successful management of the infection. Surgical resection may be an option for treating bacteremia due to BT that does not respond to antibiotics.

## CONCLUSIONS

Surgery was beneficial for both diagnostic and therapeutic purposes in this case. Resection of the intestine, a source of BT, is a treatment option when bacteremia does not respond to antibiotics.

## References

[1] Berg RD, Owens WE. Inhibition of translocation of viable Escherichia coli from the gastrointestinal tract of mice by bacterial antagonism. Infect Immun 1979; 25: 820–7. 159260 10.1128/iai.25.3.820-827.1979 PMC414521

[2] Sedman PC, Macfie J, Sagar P, et al. The prevalence of gut translocation in humans. Gastroenterology 1994; 107: 643–9. 8076751 10.1016/0016-5085(94)90110-4

[3] Kouzu K, Tsujimoto H, Kishi Y, et al. Bacterial translocation in gastrointestinal cancers and cancer treatment. Biomedicines 2022; 10: 380. 35203589 10.3390/biomedicines10020380 PMC8962358

[4] Cusumano G, Flores GA, Venanzoni R, et al. The impact of antibiotic therapy on intestinal microbiota: dysbiosis, antibiotic resistance, and restoration strategies. Antibiotics (Basel) 2025; 14: 371. 40298495 10.3390/antibiotics14040371 PMC12024230

[5] Charitos IA, Scacco S, Cotoia A, et al. Intestinal microbiota dysbiosis role and bacterial translocation as a factor for septic risk. Int J Mol Sci 2025; 26: 2028. 10.3390/ijms26052028 PMC11900423 40076650

[6] Yao S, Yagi S, Sugimoto T, et al. Occult bacteremia in living donor liver transplantation: a prospective observational study of recipients and donors. Surg Today 2024; 54: 596–605. 38072872 10.1007/s00595-023-02778-7

[7] Youssef M, Al Shurman A, Chachaty E, et al. Use of molecular typing to investigate bacterial translocation from the intestinal tract in malnourished children with Gram-negative bacteremia. Clin Microbiol Infect 1998; 4: 70–4. 11864289 10.1111/j.1469-0691.1998.tb00358.x

[8] Silva ECDF, Montalvão CR, Bonafé S. Infectious endocarditis from *Enterococcus faecalis* associated with tubular adenoma of the sigmoid colon. Case Rep Infect Dis 2017; 2017: 3095031. 28848681 10.1155/2017/3095031 PMC5564126

[9] Masood L, Müller A, Ali NZ, et al. A narrative literature review on sepsis: a primary manifestation of colorectal neoplasm. Cureus. 2023. https://www.cureus.com/articles/165298-a-narrative-literature-review-on-sepsis-a-primary-manifestation-of-colorectal-neoplasm. Accessed 2025 Aug 18. 10.7759/cureus.44803 PMC10560076 37809261

[10] Watanabe A, Harimoto N, Araki K, et al. Perioperative pancreaticoduodenectomy management strategy focusing on postoperative early drain colonization. Surg Today 2024; 54: 1067–74. 38502211 10.1007/s00595-024-02810-4

[11] Utsumi M, Inagaki M, Omoto K, et al. Clinical impact of bile culture from gallbladder in patients undergoing laparoscopic cholecystectomy for acute cholecystitis. Surg Today 2025; 55: 1655–1662. 40495023 10.1007/s00595-025-03069-z

[12] Ruoff KL, De La Maza L, Murtagh MJ, et al. Species identities of enterococci isolated from clinical specimens. J Clin Microbiol 1990; 28: 435–7. 2108992 10.1128/jcm.28.3.435-437.1990 PMC269638

[13] Nguyen P, Khicher S, Osman H, et al. A rare case of enterococcus gallinarum-associated peritonitis and literature review. Cureus 2020; 12: e12328. 33520526 10.7759/cureus.12328 PMC7839278

[14] Zhao B, Ye MS, Zheng R. Enterococcus gallinarum meningitis: a case report and literature review. BMC Infect Dis 2018; 18: 231. 29783937 10.1186/s12879-018-3151-4 PMC5963013

[15] Soranno DE, Coopersmith CM, Brinkworth JF, et al. A review of gut failure as a cause and consequence of critical illness. Crit Care 2025; 29: 91. 40011975 10.1186/s13054-025-05309-7 PMC11866815

[16] Muñoz L, Borrero M, Úbeda M, et al. Intestinal immune dysregulation driven by dysbiosis promotes barrier disruption and bacterial translocation in rats with cirrhosis. Hepatology 2019; 70: 925–38. 30414342 10.1002/hep.30349

[17] Samel S, Keese M, Kleczka M, et al. Microscopy of bacterial translocation during small bowel obstruction and ischemia in vivo – a new animal model. BMC Surg 2002; 2: 6. 12174194 10.1186/1471-2482-2-6 PMC126214

[18] Choi SH, Lee S, Kim TH, et al. Clinical features and outcomes of bacteremia caused by *Enterococcus casseliflavus* and *Enterococcus gallinarum:* Analysis of 56 Cases. Clin Infect Dis 2004; 38: 53–61. 14679448 10.1086/380452

[19] Kraszewska Z, Skowron K, Kwiecińska-Piróg J, et al. Antibiotic resistance of Enterococcus spp. Isolated from the urine of patients hospitalized in the University Hospital in North-Central Poland, 2016–2021. Antibiotics (Basel) 2022; 11: 1749. 36551406 10.3390/antibiotics11121749 PMC9774570

[20] Cetinkaya Y, Falk P, Mayhall CG. Vancomycin-resistant enterococci. Clin Microbiol Rev 2000; 13: 686–707. 11023964 10.1128/cmr.13.4.686-707.2000 PMC88957

[21] Tominaga T, Oikawa M, Takeshita H, et al. Successful colectomy for hemorrhagic colitis with hemolytic uremic syndrome and acute encephalopathy due to *Escherichia coli* O157 infection. Case Rep Gastroenterol 2014; 8: 82–8. 24803891 10.1159/000360846 PMC3999577

[22] Mizukami A, Furuya S, Takiguchi K, et al. Intraoperative indocyanine green fluorescence for precise resection of nonocclusive mesenteric ischemia: a case report and diagnostic considerations based on pathology findings. Surg Case Rep 2024; 10: 230. 39365410 10.1186/s40792-024-02024-3 PMC11452368

[23] Tateno K, Motegi Y, Ogawa H, et al. Gangrenous ischemic colitis localized to the cecum: a case report. Surg Case Rep 2023; 9: 9. 36689043 10.1186/s40792-023-01587-x PMC9871088

